# Efficacy and Safety of Injectable and Oral Antibiotics in Treating Gonorrhea: A Systematic Review and Network Meta-Analysis

**DOI:** 10.3390/jcm8122182

**Published:** 2019-12-11

**Authors:** Jiaru Yang, Subhash Dhital, Thomas Naderer

**Affiliations:** Department of Biochemistry & Molecular Biology, Biomedicine Discovery Institute, Monash University, Clayton, VIC 3800, Australia

**Keywords:** *Neisseria gonorrhoeae*, gonorrhea, antibiotics, treatment, network meta-analysis

## Abstract

Gonorrhea is the second most frequently reported sexually transmitted infectious disease of bacterial origin in the world. Current empiric therapies rely on broad-spectrum antibiotics. However, treatment options are becoming limited due to the rise of drug-resistant gonorrhea. To control the rise of drug-resistant gonorrhea and to identify alternative treatment options, clinicians will have to increasingly rely on experimental evidence for the treatment of gonorrhea patients. Thus, we performed a systematic review and network meta-analysis of all randomized clinical trials about the efficacy and safety of various antibiotic regimens in adults with gonorrhea. We searched all references in Embase and PubMed from the date of their inception to January 2019, and then an updated search was performed in March 2019. Of the 28,843 identified references, 44 fulfilled our selection criteria. We used a network meta-analysis based on a frequentist approach to evaluate the efficacy and safety of 12 injectable and 11 oral antibiotics. The efficacy of treatments was ranked by *p* score and inconsistency was assessed by a back-calculation method. Certainty of evidence was evaluated by the GRADE system. For injectable drugs, there was no difference in efficacy between a reference antibiotic and other drugs. However, ceftriaxone had significantly better efficacy than cefuroxime (OR, 12.03; 95% CI 3.73–38.79), cephaloridine (OR, 42.41; 95% CI 8.77–205.07), kanamycin (OR, 5.45; 95% CI 1.25–23.70), penicillin (OR, 13.11; 95% CI 4.48–38.37), and spectinomycin (OR, 4.70; 95% CI 1.62–13.62). Thus, ceftriaxone was the most effective injectable drug (*p* score of 0.924). As for oral drugs, azithromycin was the most effective compound (*p* score of 0.8633). There were no significant differences in safety between injectable and oral treatments. In our systematic review of randomized controlled trials, we found azithromycin and ceftriaxone to be the most effective antibiotics for the treatment of gonorrhea. This is in line with current guidelines which recommend a combination therapy of azithromycin and ceftriaxone for the treatment of gonorrhea due to increased antimicrobial resistance. Our analysis identified gentamicin and ofloxacin as alternative therapeutics to treat drug-resistant gonorrhea.

## 1. Introduction

*Neisseria gonorrhoeae* is a Gram-negative diplococcus and the etiological agent of gonorrhea, a sexually transmitted infection (STI). There are 78–106 million new cases of gonorrhea worldwide each year, with a global incidence rate of 19 per 1000 females and 24 per 1000 males [1], and the rate of transmission has doubled in the last five years in several countries, including Australia [2]. 

*N. gonorrhoeae* evades host immune responses and is able to establish infection at the mucosal surfaces of the urogenital tract, pharynx, and rectum. In many cases, *N. gonorrhoeae* infections remain asymptomatic, which promotes dissemination to other individuals during sexual intercourse. However, gonorrhea can develop into several inflammatory diseases including urethritis, endometritis, salpingitis, epididymitis, and pelvic inflammatory disease (PID). PID can lead to infertility and still births, whereas urethritis in males results in painful micturition and pain or tenderness in the testicles [3,4]. Previously, antibiotics such as penicillin have been highly effective in treating gonorrhea. However, antimicrobial resistance has spread rapidly due to the emergence of several mechanisms, including mutations of drug targets, increased expression of efflux pumps, and the acquisition of antibiotic-degrading enzymes [1,5,6,7]. As with other drug-resistant infections, antibiotics use has further driven antimicrobial resistance in *N. gonorrhoeae* [8,9,10]. In the absence of new antibiotics, current treatment guidelines recommend dual therapy based on azithromycin and ceftriaxone. However, resistance to all currently used drugs circulates in the gonococcal population, and dual therapy has so far failed to reduce gonorrhea infection rates. 

Therefore, there is a need to develop an evidence-based guideline for the use of antibiotics to better control or manage drug-resistant gonorrhea. In the absence of new drugs, an understanding of the efficacy and safety of current antibiotics will be able to guide physicians in the treatment of gonorrhea. To make reasonable and effective clinical decisions, physicians will depend on a systematic and quantitative evaluation of current antibiotics in the treatment of gonorrhea. Therefore, we performed a systematic review and network meta-analysis of all randomized clinical trials about the efficacy and safety of antibiotic regimens in adults with gonorrhea. 

## 2. Methods

### 2.1. Reporting Guideline and Certainty of Evidence

This network meta-analysis (NMA) was conducted in accordance with the Preferred Reporting Items for Systematic Reviews and Meta-Analyses (PRISMA) extension statement for systematic reviews incorporating network meta-analyses of health care interventions [11]. 

For assessing the certainty of evidence of this NMA, we used the Grading of Recommendations Assessment, Development and Evaluation (GRADE) system [12,13,14,15,16,17]. GRADE offers a system for rating the quality of evidence in systematic reviews [18]. The GRADE system evaluates evidence in four levels of quality—high, moderate, low, and very low. High quality (⊕⊕⊕⊕) means that we are very confident that the true effect lies close to that of the estimate of the effect. Moderate quality (⊕⊕⊕O) indicates that we are moderately confident in the effect estimate—the true effect is likely to be close to the estimate of the effect, but there is a possibility that it is substantially different. Low quality (⊕⊕OO) signifies that our confidence in the effect estimate is limited—the true effect may be substantially different from the estimate of the effect. Very low quality (⊕OOO) indicates that we have very little confidence in the effect estimate—the true effect is likely to be substantially different from the estimate of effect [17].

### 2.2. Search Strategies and Inclusion Criteria

We searched all references in Embase and PubMed from the date of their inception to January 2019, and then an updated search was performed in March 2019. The search terms were “gonorrhea”, “randomized controlled trial”, “controlled clinical trial”, “random allocation”, “double-blind”, “single-blind”, “survival”, “treatment”, and “therapy”, combined with a list of antibiotics. Initially, we used 10 antibiotics, and repeated the search with additional 16 antibiotics, including azithromycin, cefixime, cefpodoxime, ceftriaxone, ciprofloxacin, gentamicin, penicillin, sitafloxacin, spectinomycin, tetracycline, amoxicillin, ampicillin, cefotaxime, cefoxitin, cefuroxime, cephaloridine, doxycycline, enoxacin, fleroxacin, kanamycin, minocycline, norfloxacin, ofloxacin, pefloxacin, solithromycin, and zoliflodacin.

All included references had to be written in English and had to be randomized controlled trials (RCTs) comparing the efficacy and safety of antibiotics for the treatment of gonorrhea. In addition, we only included references that used monotherapy of oral or injectable drugs. We excluded studies on RCTs with a different method of antibiotic delivery, as this could affect the efficacy of a particular drug. Patients in RCTs had to be adults. More importantly, all patients in RCTs had to have been diagnosed positive for *N. gonorrhoeae* infection using established techniques including microscopy, Gram stain, bacteriological culture, or oxidase reaction. All included studies were independently scanned by two investigators. Any disagreements with each assessment were resolved by discussing the issues until a consensus was reached.

### 2.3. Data Extraction

From each reference, we extracted data on patients, interventions, and outcomes. For patients, we collected the total number of patients in each RCT, as well as the age and the gender. For interventions, we collected the antibiotic agent, dosage, and length of treatment. For outcomes, we determined the efficacy and safety of drugs. For this, the number of patients who were completely recovered after treatment and the number of patients who reported adverse reactions after or during treatment were recorded. Efficacy was then determined by the response rate of treatments based on the total number of patients who were completely recovered and the total number of patients in each RCT. Safety referred to the incidence of drug side-effects, which was calculated by the total number of patients who reported adverse reactions after or during treatment and the total number of patients in each RCT. 

Patients who were absent in the follow-up interview were excluded from our study.

### 2.4. Statistical Analysis

We estimated the risk of bias of all included references in accordance with to the Cochrane Collaboration’s Tool for Assessing Risk of Bias in Randomised Trials [19]. 

Network meta-analysis is a generalization of pairwise meta-analysis that can compare all pairs of treatments within a number of treatments for the same condition [20,21]. Thus, we used NMA to evaluate the efficacy and safety of antibiotics for the treatment of gonorrhea [22,23]. We then used random effects and consistency model to analyze all extracted data. Odds ratios (ORs) served as an indicator for evaluating two primary outcomes (efficacy and safety) in this NMA.

Inconsistency is another important indicator that needs to be assessed in an NMA. We compared estimates of the effect of direct and indirect evidence to assess inconsistency of NMA [24]. Inconsistency was the existence in an NMA if the *p*-value was less than 0.05. We used the back-calculation method to derive indirect estimates from direct pairwise comparisons and network estimates [25] and then assessed the difference of estimates between direct and indirect evidence to check the inconsistency of this NMA.

To rank the efficacy and safety of antibiotics, we used the *p* score as an indicator that is solely based on the point estimates and standard errors of the network estimates. They measure the extent of certainty that a treatment is better than another treatment, averaged over all competing treatments [26]. The *p* score is measured on a scale from 0 (worst) to 1 (best), which means if a treatment is better than others, its *p* score will also be large. 

All analysis was conducted by NETMETA package [22] based on R (version 3.5.2) and STATA (version 14.0). 

## 3. Results

### 3.1. Literature Search Process and Study Characteristics

**Figure 1:**
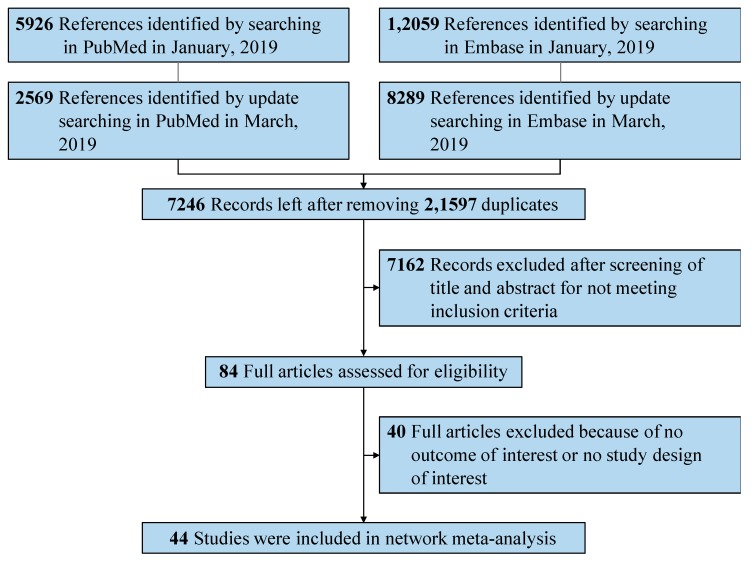
Preferred Reporting Items for Systematic Reviews and Meta-Analyses (PRISMA) flowchart of search strategy and study selection.

We analyzed a total of 28,843 references from Embase and PubMed. After independent manual assessment by two investigators, we identified 44 publications that met the inclusion criteria (Figure 1). Based on these publications, we performed the following analysis.

All included references were published during 1970–1997, which involved 10,204 patients. Twenty-five studies used intramuscular injection monotherapy that involved 6422 patients and compared the efficacy of 12 different drugs [27,28,29,30,31,32,33,34,35,36,37,38,39,40,41,42,43,44,45,46,47,48,49,50,51]. Nineteen of these studies reported adverse reactions in 569 patients and the safety of 11 drugs was assessed [27,28,29,30,31,32,33,34,36,37,38,39,40,41,44,45,46,49,50]. Similarly, 19 studies focused on oral monotherapy [51,52,53,54,55,56,57,58,59,60,61,62,63,64,65,66,67,68,69,70]. These studies of oral drugs involved 3782 patients, and compared the efficacy of 11 antibiotics. Thirteen of these studies reported side-effects and compared the safety of eight drugs in a total of 219 patients [52,53,54,55,56,57,58,59,60,61,64,65,66].

**Figure 2:**
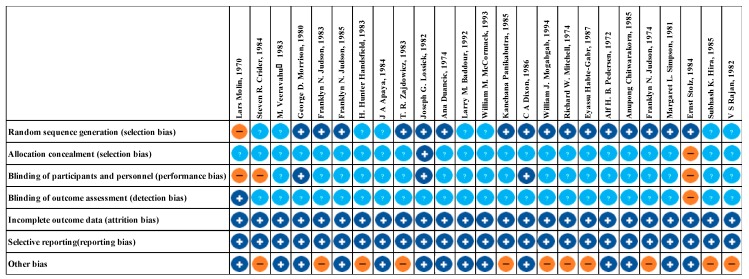
Risk of bias assessment summary of injectable antibiotics. + low risk of bias. − high risk of bias. ? unclear risk of bias.

**Figure 3:**
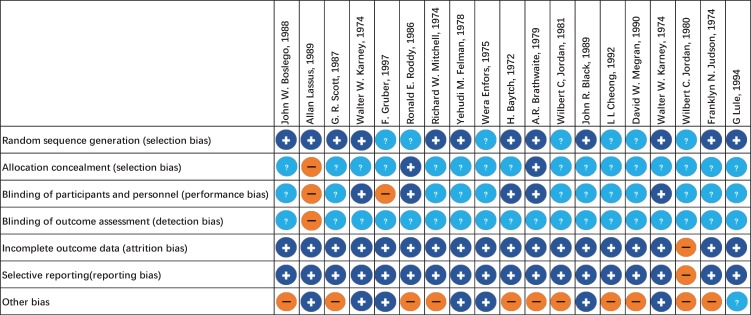
Risk of bias assessment summary of oral antibiotics. + low risk of bias. − high risk of bias. ? unclear risk of bias.

The evaluation of risk of bias for RCTs of injectable and oral antibiotic are presented in Figure 2 and Figure 3, respectively. We determined bias to assess the quality of RCTs based on seven principles. We concluded that at least 89% of these studies were of sufficient quality (Figure 2 and Figure 3).

### 3.2. Pairwise Efficacy of Antibiotics

Significant differences in efficacy were present among both injectable and oral drugs.

**Figure 4:**
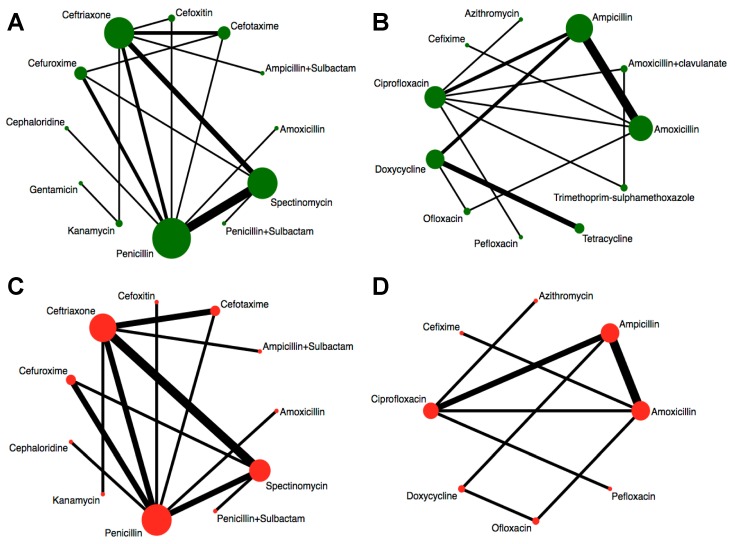
Network graphs of antibiotics. Width of the lines is proportional to the number of trials comparing every pair of treatments. Size of circles is proportional to the number of patients. (**A**) Network graph of injectable drugs for efficacy. (**B**) Network graph of oral drugs for efficacy. (**C**) Network graph of injectable drugs for safety. (**D**) Network graph of oral drugs for safety.

**Figure 5:**
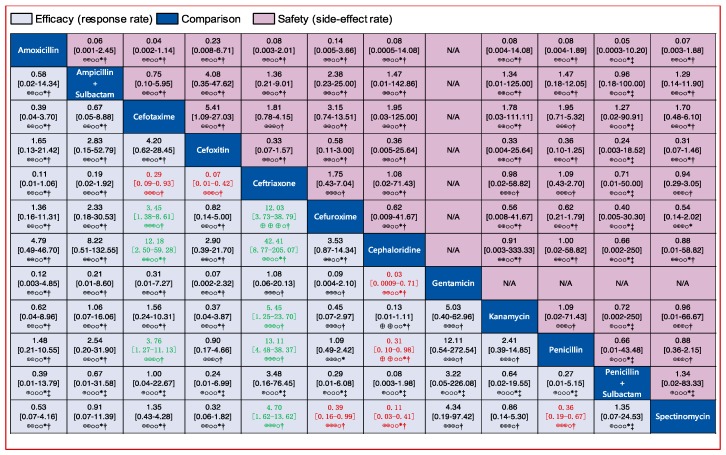
Pairwise comparisons of the efficacy and safety of 12 injectable antibiotics. Drugs are reported in alphabetical order. Data are ORs and 95% CIs in each grid. For efficacy, each grid shows the result of efficacy comparison of column-defining drug versus row-defining drug. If both the OR and 95% CI are higher than 1 it indicates that the column-defining drug’s efficacy is better than that of the row-defining drug. For safety, if both the OR and 95% CI are higher than 1 it indicates that the column-defining drug’s safety is worse than that of the row-defining drug. In other words, it means that the column-defining drug more easily induces adverse reactions. To obtain ORs for comparisons in the opposite direction, reciprocals should be taken. Significant results (the range of 95% CI does not include 1) are in red and green. The certainty of the evidence (according to Grading of Recommendations Assessment, Development and Evaluation (GRADE)) is incorporated in this figure.⊕⊕⊕⊕ High quality. ⊕⊕⊕O Moderate quality. ⊕⊕OO Low quality. ⊕OOO Very low quality. ^†^ Downgraded once for study limitations (risk of bias). ^‡^ Downgraded twice for serious study limitations (risk of bias). * Downgraded once for imprecision. ¶ Downgraded twice for serious imprecision.

**Figure 6:**
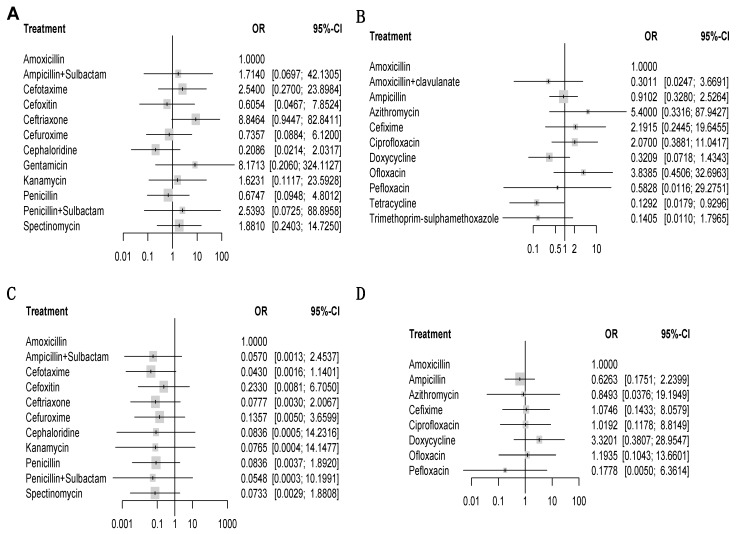
Forest plots of network meta-analysis of all trials for efficacy and safety. Antibiotics versus amoxicillin (reference drug). OR = odds ratio. CI = confidence interval. Colorful rectangles represent the ORs. Black lines represent the range of 95% CIs. For efficacy, an OR higher than 1 means the efficacy of a certain drug is better than that of amoxicillin. For safety, an OR higher 1 means the safety of a certain drug is worse than that of amoxicillin. The differences in efficacy or safety can be regarded as a significant when the range of the 95% CI does not include 1. In other words, it means there is significant difference between a certain drug and amoxicillin. (**A**) comparisons of injectable drugs for efficacy. (**B**) Comparisons of oral drugs for efficacy. (**C**) Comparisons of injectable drugs for safety. (**D**) Comparisons of oral drugs for safety.

We assessed the efficacy of 12 injectable antibiotics. The three most widely used antibiotics, in terms of patients treated and number of clinical trials, were penicillin (1603 patients treated, 14 clinical trials), ceftriaxone (683 patients treated, 9 clinical trials), and spectinomycin (821 patients treated, 10 clinical trials) (Figure 4A). The results of pairwise comparison are indicated by the odds ratios (ORs) and 95% confidence intervals in Figure 5. We used amoxicillin as reference as it is used as injectable and oral antibiotic. There was no difference in efficacy between amoxicillin and other injectable drugs (Figure 5 and Figure 6A). In contrast, the efficacy of cefotaxime was significantly better than that of cefuroxime, cephaloridine, and penicillin. The OR of cefotaxime vs. cefuroxime was 3.45 and the 95% CI was 1.38–8.61. The OR of cefotaxime vs. cephaloridine was 12.18 and the 95% CI was 2.50–59.28. The OR of cefotaxime vs. penicillin was 3.76 and the 95% CI was 1.27–11.13 (Figure 5). However, ceftriaxone showed significantly better efficacy than most other antibiotics including cefuroxime, cephaloridine, kanamycin, penicillin, and spectinomycin. The OR of ceftriaxone vs. cefuroxime was 12.03 and the 95% CI was 3.73–38.79. The OR of ceftriaxone vs. cephaloridine was 42.41 and the 95% CI was 8.77–205.07. The OR of ceftriaxone vs. kanamycin was 5.45 and the 95% CI was 1.25–23.70. The OR of ceftriaxone vs. penicillin was 13.11 and the 95% CI was 4.48–38.37. The OR of ceftriaxone vs. spectinomycin was 4.70 and the 95% CI was 1.62–13.62 (Figure 5). The latter was more effective than cefuroxime, cephaloridine, and penicillin (Figure 5, labelled red). Cefotaxime and cefoxitin were less efficient than ceftriaxone (Figure 5, labelled red). There were no significant differences in efficacy among the remaining comparisons. The certainty of evidence of these results were moderate or low, but most of them were moderate (Figure 5).

**Figure 7:**
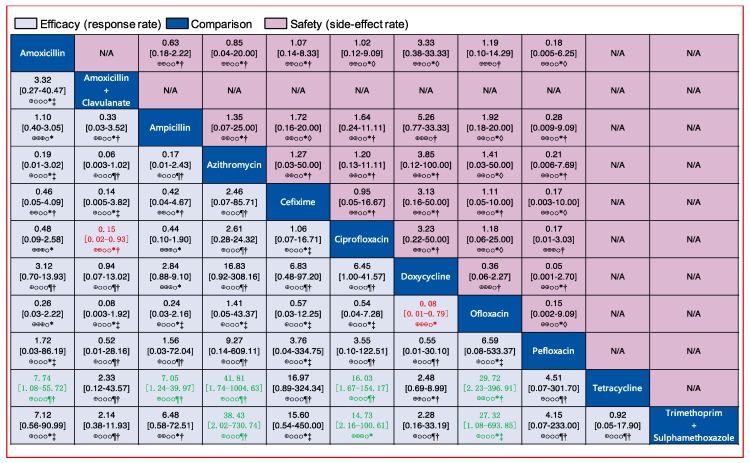
Pairwise comparisons for the efficacy and safety of the 11 oral antibiotics. Drugs are reported in alphabetical order. Data are ORs and 95% CIs in each grid. For efficacy, each grid shows the result of efficacy comparison of column-defining drug versus row-defining drug. If both the OR and 95% CI are higher than 1 it indicates that the column-defining drug’s efficacy is better than that of row-defining drug. For safety, if both the OR and 95% CI are higher than 1 it indicates that the column-defining drug’s safety is worse than that of the row-defining drug. In other word, it means that the column-defining drug more easily induces adverse reactions. To obtain ORs for comparisons in the opposite direction, reciprocals should be taken. Significant results (the range of 95% CI does not include 1) are in red and green. The certainty of the evidence (according to GRADE) is incorporated in this figure. ⊕⊕⊕⊕ High quality. ⊕⊕⊕O Moderate quality. ⊕⊕OO Low quality. ⊕OOO Very low quality. ^†^ Downgraded once for study limitations (risk of bias). ^‡^ Downgraded twice for serious study limitations (risk of bias). * Downgraded once for imprecision. ¶ Downgraded twice for serious imprecision. ◊ Downgraded once for heterogeneity.

Similarly, we analyzed the efficacy of 11 oral drugs whereby ampicillin (990 patients treated, 9 clinical trials), amoxicillin (550 patients treated, 7 clinical trials), and ciprofloxacin (302 patients treated, 7 clinical trials) were the most widely studied antibiotics (Figure 4B). The results of comparison for the efficacy of oral drugs are displayed on the Figure 7 and Figure 6B. Amoxicillin, ampicillin, azithromycin, ciprofloxacin, and ofloxacin had better efficacy than tetracycline (Figure 7, labelled green). Moreover, azithromycin, ciprofloxacin, and ofloxacin were more effective than trimethoprim plus sulfamethoxazole (Figure 7, labelled green). In contrast, amoxicillin plus clavulanate and doxycycline were significantly less effective than ciprofloxacin (OR, 0.15; 95% CI 0.02–0.93) and ofloxacin (OR, 0.08; 95% CI 0.01–0.79), respectively (Figure 7, labelled red). Nevertheless, we should note that the certainties of evidence of these results were moderate, low, or very low (most of them were very low; Figure 7). As for the remaining comparisons, there were no significant differences in efficacy among them. 

### 3.3. Safety of Antibiotics

The incidence of adverse reactions (side-effect) was used to evaluate the safety of the drugs. The total number of patients who had adverse reactions comprised 7.72% of total patients who received treatments. Adverse reactions occurred on various ages of both male and female patients and were involved many parts of body, while most them were mild. Commonly reported side-effects included vomiting, dizziness, rash, pruritus, somnolence, edema, serum sickness, urticaria, vertigo, nausea, dyspepsia, abdominal pain, diarrhea, headache, gastrointestinal disturbance, allergic reaction, and chills. For injectable drugs, another common reaction was pain at the injection site. 

Comparisons of injectable and oral drugs for safety are displayed in Figure 4C,D. Assessments of the safety of injectable and oral drugs are displayed in Figure 5 and Figure 7, and Figure 6C,D, respectively. However, there were no significant differences in safety among those treatments. 

### 3.4. Ranking of Antibiotic Efficacy against Gonorrhea

**Figure 8:**
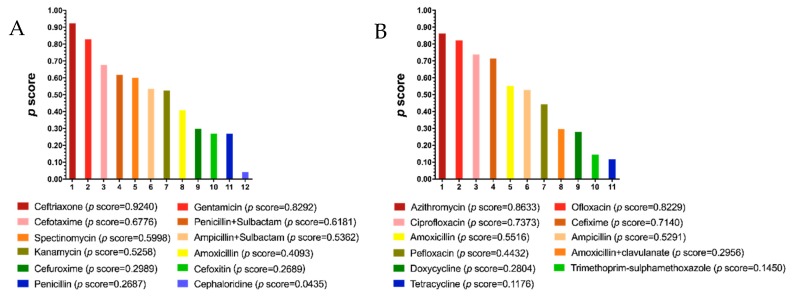
Ranking of efficacy of injectable (**A**) and oral (**B**) antibiotics. The *p* score is an indicator of efficacy ranking. A higher *p* score indicates a higher efficacy.

Next, we used the calculated *p* scores to rank the efficacy of antibiotics, which provides a reference for clinicians. Amongst injectable drugs, ceftriaxone had the highest efficacy with a *p* score of 0.9240 (Figure 8A). As for oral drugs, the *p* score of azithromycin was 0.8633, which showed the highest efficacy (Figure 8B). Besides ceftriaxone and azithromycin, we identified gentamicin (*p* = 0.8292) and ofloxacin (*p* = 0.8229) as having high efficacy for the treatment of gonorrhea (Figure 8A,B). The ranking of drugs’ safety could not be provided due to the lack of differences among those comparisons of safety (Figure 5 and Figure 7).

Moreover, we tested inconsistency for all comparisons. The results are displayed in Table 1. Most *p*-values of these comparisons were greater than 0.05, which means inconsistency was not present and they were of good quality. The exception among injectable drugs was penicillin vs. spectinomycin, with a *p*-value of 0.047; among oral drugs the exceptions were cefuroxime vs. penicillin and oral cefuroxime vs. spectinomycin, both with *p*-values of 0.0014.

### 3.5. Antibiotic Efficacy Based on Gonorrhea Infection Sites

*N. gonorrhoeae* can infect a range of mucosal surfaces, and some antibiotics fail to clear infection sites, particularly within the oral cavity. The reported studies included infections of four different sites—rectum, urethra, endocervix, and pharynx. For oral drugs, there was no significant difference in efficacy for treating different infection sites. In contrast, injectable ceftriaxone was more effective than spectinomycin for treating pharyngeal infection (OR, 20.00; 95% CI 3.37–118.62). Cefotaxime was better than cefuroxime for treating urethral infection (OR, 3.40; 95% CI 1.73–6.71). The remaining injectable drugs did not display significant differences in efficacy for treating different infection sites. 

## 4. Discussion

This network meta-analysis evaluated the efficacy and safety of 22 treatment regimens for treating gonorrhea via analyzing pooled effect size of 44 RCTs, which involved 10,204 patients.

By analyzing the odds ratios in each RCT, irrespective of therapeutic duration and dosage, we found that there were differences in efficacy among various treatments. For injectable antibiotics, ceftriaxone (*p* score 0.924) had better efficacy than other drugs, especially cefuroxime, cephaloridine, kanamycin, penicillin, and spectinomycin. Besides ceftriaxone, cefotaxime’ efficacy was better than other antibiotics, especially cefuroxime, cephaloridine, and penicillin. On the contrary, cephaloridine could be regarded as the least effective injectable drug, with a *p* score of 0.0435. Our analysis identified gentamicin as an effective alternative to current cephalosporins such as ceftriaxone and cefotaxime. This is in line with a recent report showing that combination therapy of gentamicin and azithromycin is highly effective against uncomplicated gonorrhea [71].. A more recent randomized non-inferiority trial concluded that gentamicin is effective in clearing genital gonorrhea, but less so for pharyngeal and rectal infections [72]. Gentamicin resistance in culture has not been reported in clinical isolates of gonorrhea [73], and little is known about gentamicin resistance in the clinic due to its limited use. Our analysis showed that gentamicin had the same safety profile as ceftriaxone, which further warrants the use of gentamicin as potential alternative for the treatment of gonorrhea.

For oral drugs, azithromycin was the most effective treatment (*p* score 0.8633), whereas tetracycline had the worst efficacy score (*p* score 0.1176). We identified ofloxacin to be highly effective (*p* score 0.8229). These results indicate ofloxacin as a potential alternative antibiotic. In addition, our analysis indicates ciprofloxacin as an effective antibiotic. Currently, ofloxacin and ciprofloxacin are not recommended as first-line treatment options against gonorrhea. Our results suggest that in the absence of effective azithromycin therapy, physicians may consider ofloxacin and ciprofloxacin for the treatment of gonorrhea. This is not withstanding any new antibiotics, such as solithromycin and zoliflodacin, that are currently being trialed in the clinic. 

As for inconsistency in this NMA, most drug comparisons did not present inconsistency (their *p*-values were greater than 0.05). However, there was a significant difference in the comparison of injectable penicillin and spectinomycin because the *p*-value was 0.047 (less than 0.05), which means inconsistency existed between direct and indirect evidence for the efficacy of these two drugs (Table 1). Similarly, inconsistency also existed in comparisons for the safety of injectable drugs—that is, cefuroxime vs. penicillin and cefuroxime vs. spectinomycin (Table 1). Based on the GRADE system’s advice [17] for dealing with inconsistency, we needed to evaluate the results of our comparison for the efficacy or safety of these drugs in detail.

In the comparison of penicillin versus spectinomycin, the direct evidence proportion was 85%. Besides, the estimate of the effect of penicillin versus spectinomycin derived from direct evidence gave an OR of 0.27 and the 95% CI was 0.14–0.54, while from indirect evidence the OR was 1.58 and the 95% CI was 0.32–7.72. Obviously, the 95% CI of indirect evidence was much wider than that of direct evidence, which means that the direct evidence had better precision. On the other hand, there were five RCTs that involved 1122 patients providing direct evidence for comparison, which is more than that of indirect evidence (945 patients). Thus, we suggest accepting the results of the comparison derived from direct evidence because it was more reliable.

In comparisons of cefuroxime vs. penicillin, the direct evidence proportion was 85% and the estimate of its effect gave an OR of 0.87 and the 95% CI was 0.28–2.69, which was more precise than that of indirect evidence (OR, 205.53; 95% CI 8.80–4799.16). Moreover, the direct evidence involved 1074 patients, which is more than the number of patients in the indirect evidence (969 patients). Hence, the direct evidence was more reliable than indirect evidences, and we suggest accepting the results of the comparison derived from direct evidence. 

In contrast, the indirect evidence of cefuroxime vs. spectinomycin was more reliable than the direct evidence. This is because the estimate of the effect for direct evidence gave an OR of 153.63 and the 95% CI was 7.57–3116.66, which is more imprecise than indirect evidence (OR, 0.65; 95% CI 0.15–2.81). Moreover, the direct evidence proportion was only 19%. More importantly, direct evidence only involved 365 patients which was much less than the number of patients accounted for in the indirect evidence (1678 patients). 

Regarding the limitations of this NMA, the most important limitation was that some RCTs did not involve sufficient number of patients, so the estimate effect of some treatments had a wide confidence interval, indicating that the results of those comparisons were low in precision and reliability. Furthermore, we did not focus on therapy duration or drug dosage when we conducted this NMA, which may beset clinicians’ decisions. Some of the included RCTs had a low level in the assessment of risk of bias, which may lead to the results of the comparison for efficacy or safety derived from these RCTs being different from the real efficacy or safety of the drugs. Moreover, most results of the comparisons had low or very low certainty of evidence, which also implied that large differences may exist among their NMA results and real efficacy or safety. Importantly, the majority of RCTs were performed between 1970 and 1997, with only limited numbers in more recent years. Given that antimicrobial resistance has developed rapidly over the last few decades, our analysis may not reflect current antimicrobial trends in the clinic. As such, the reported efficacy of antibiotics are likely overestimated, particularly in the case of penicillin, which further emphasizes the urgency of identifying new treatment options against gonorrhea. 

Overall, we identified three more effective antibiotics (gentamycin, ofloxacin, and ciprofloxacin) for the treatment of gonorrhea and recommend RCTs to more adequately capture the efficacy of current therapeutic interventions. 

## 5. Conclusions

For gonorrhea, empirical therapy is frequent. Current guidelines include dual therapy with azithromycin and ceftriaxone, largely because of the rapid rise of resistance towards monotherapy and other antibiotics. According to our analysis, ceftriaxone appeared to be the best treatment among 12 different injectable antibiotic regimens. Apart from ceftriaxone, gentamicin and cefotaxime therapy may be appropriate alternative treatment options. Similarly, our analysis identified azithromycin as the most effective antibiotic for treating gonorrhea. Besides azithromycin, ofloxacin and ciprofloxacin appear to be effective alternative drugs. Notwithstanding, resistance to ofloxacin and ciprofloxacin have been reported, whereas there is limited information on gentamicin in treating gonorrhea. The majority of published clinical trials are based on monotherapy, and it would be interesting to test other dual or multiple therapies to identify urgently needed alternative options against gonorrhea.

## Figures and Tables

**Table 1:** Assessment of inconsistency. *p*-value > 0.05 indicates there was no inconsistency among direct and indirect comparisons; a *p*-value < 0.05 indicates inconsistency among direct and indirect comparisons (in bold).

Injectable Drugs		Oral Drugs		Side-Effect Injectable Drugs		Side-Effect of Oral Drugs	
Comparison	*p*-Value	Comparison	*p*-Value	Comparison	*p*-Value	Comparison	*p*-Value
Penicillin vs. Spectinomycin	**0.047**	Amoxicillin vs. Ampicillin	0.5964	Cefotaxime vs. Ceftriaxone	0.6689	Amoxicillin vs. Ampicillin	0.5016
Cefuroxime vs. Spectinomycin	0.3831	Amoxicillin vs. Ciprofloxacin	0.5218	Cefotaxime vs. Penicillin	0.6689	Amoxicillin vs. Ciprofloxacin	0.9755
Cefuroxime vs. Penicillin	0.4805	Amoxicillin vs. Ofloxacin	0.967	Ceftriaxone vs. Penicillin	0.8017	Amoxicillin vs. Ofloxacin	0.3817
Ceftriaxone vs. Spectinomycin	0.0959	Ampicillin vs. Ciprofloxacin	0.5218	Ceftriaxone vs. Spectinomycin	0.8415	Ampicillin vs. Ciprofloxacin	0.9755
Ceftriaxone vs. Penicillin	0.4123	Ampicillin vs. Doxycycline	0.967	Cefuroxime vs. Penicillin	**0.0014**	Ampicillin vs. Doxycycline	0.3817
Cefoxitin vs. Penicillin	0.2379	Doxycycline vs. Ofloxacin	0.967	Cefuroxime vs. Spectinomycin	**0.0014**	Doxycycline vs. Ofloxacin	0.3817
Cefoxitin vs. Ceftriaxone	0.2379			Penicillin vs. Spectinomycin	0.055		
Cefotaxime vs. Penicillin	0.5115						
Cefotaxime vs. Cefuroxime	0.9661						
Cefotaxime vs. Ceftriaxone	0.6748						

## References

[1] Unemo M., Jensen J.S. (2017). Antimicrobial-resistant sexually transmitted infections: Gonorrhoea and Mycoplasma genitalium. Nat. Rev. Urol..

[2] Suay-Garcia B., Perez-Gracia M.T. (2018). Future Prospects for Neisseria gonorrhoeae Treatment. Antibiotics.

[3] Escobar A., Rodas P.I., Acuna-Castillo C. (2018). Macrophage-Neisseria gonorrhoeae Interactions: A Better Understanding of Pathogen Mechanisms of Immunomodulation. Front. Immunol..

[4] Costa-Lourenco A., Barros Dos Santos K.T., Moreira B.M., Fracalanzza S.E.L., Bonelli R.R. (2017). Antimicrobial resistance in Neisseria gonorrhoeae: History, molecular mechanisms and epidemiological aspects of an emerging global threat. Braz. J. Microbiol..

[5] Skerlev M., Culav-Koscak I. (2014). Gonorrhea: New challenges. Clin. Derm..

[6] Fifer H., Natarajan U., Jones L., Alexander S., Hughes G., Golparian D., Unemo M. (2016). Failure of dual antimicrobial therapy in treatment of gonorrhea. N. Engl. J. Med..

[7] Singh A.E., Gratrix J., Martin I., Friedman D.S., Hoang L., Lester R., Metz G., Ogilvie G., Read R., Wong T. (2015). Gonorrhea Treatment Failures With Oral and Injectable Expanded Spectrum Cephalosporin Monotherapy vs Dual Therapy at 4 Canadian Sexually Transmitted Infection Clinics, 2010-2013. Sex. Transm. Dis..

[8] Olesen S.W., Barnett M.L., MacFadden D.R., Brownstein J.S., Hernández-Díaz S., Lipsitch M., Grad Y.H. (2018). The distribution of antibiotic use and its association with antibiotic resistance. eLife.

[9] Albrich W.C., Monnet D.L., Harbarth S. (2004). Antibiotic selection pressure and resistance in Streptococcus pneumoniae and Streptococcus pyogenes. Emerg. Infect. Dis..

[10] Sánchez-Busó L., Golparian D., Corander J., Grad Y.H., Ohnishi M., Flemming R., Parkhill J., Bentley S.D., Unemo M., Harris S.R. (2019). The impact of antimicrobials on gonococcal evolution. Nat. Microbiol..

[11] Hutton B., Salanti G., Caldwell D.M., Chaimani A., Schmid C.H., Cameron C., Ioannidis J.P., Straus S., Thorlund K., Jansen J.P. (2015). The PRISMA extension statement for reporting of systematic reviews incorporating network meta-analyses of health care interventions: Checklist and explanations. Ann. Intern. Med..

[12] Guyatt G.H., Oxman A.D., Kunz R., Brozek J., Alonso-Coello P., Rind D., Devereaux P.J., Montori V.M., Freyschuss B., Vist G. (2011). GRADE guidelines 6. Rating the quality of evidence--imprecision. J. Clin. Epidemiol..

[13] Guyatt G.H., Oxman A.D., Kunz R., Woodcock J., Brozek J., Helfand M., Alonso-Coello P., Falck-Ytter Y., Jaeschke R., Vist G. (2011). GRADE guidelines: 8. Rating the quality of evidence--indirectness. J. Clin. Epidemiol..

[14] Guyatt G.H., Oxman A.D., Kunz R., Woodcock J., Brozek J., Helfand M., Alonso-Coello P., Glasziou P., Jaeschke R., Akl E.A. (2011). GRADE guidelines: 7. Rating the quality of evidence--inconsistency. J. Clin. Epidemiol..

[15] Guyatt G.H., Oxman A.D., Montori V., Vist G., Kunz R., Brozek J., Alonso-Coello P., Djulbegovic B., Atkins D., Falck-Ytter Y. (2011). GRADE guidelines: 5. Rating the quality of evidence--publication bias. J. Clin. Epidemiol..

[16] Guyatt G.H., Oxman A.D., Vist G., Kunz R., Brozek J., Alonso-Coello P., Montori V., Akl E.A., Djulbegovic B., Falck-Ytter Y. (2011). GRADE guidelines: 4. Rating the quality of evidence—study limitations (risk of bias). J. Clin. Epidemiol..

[17] Puhan M.A., Schunemann H.J., Murad M.H., Li T., Brignardello-Petersen R., Singh J.A., Kessels A.G., Guyatt G.H., Group G.W. (2014). A GRADE Working Group approach for rating the quality of treatment effect estimates from network meta-analysis. BMJ.

[18] Guyatt G., Oxman A.D., Akl E.A., Kunz R., Vist G., Brozek J., Norris S., Falck-Ytter Y., Glasziou P., DeBeer H. (2011). GRADE guidelines: 1. Introduction—GRADE evidence profiles and summary of findings tables. J. Clin. Epidemiol..

[19] Higgins J.P., Altman D.G., Gotzsche P.C., Juni P., Moher D., Oxman A.D., Savovic J., Schulz K.F., Weeks L., Sterne J.A. (2011). The Cochrane Collaboration’s tool for assessing risk of bias in randomised trials. BMJ.

[20] Dias S., Welton N.J., Sutton A.J., Ades A. (2013). Evidence synthesis for decision making 1: Introduction. Med. Decis. Mak..

[21] Dias S., Welton N.J., Sutton A.J., Caldwell D.M., Lu G., Ades A. (2013). Evidence synthesis for decision making 4: Inconsistency in networks of evidence based on randomized controlled trials. Med. Decis. Mak..

[22] Rücker G. (2012). Network meta-analysis, electrical networks and graph theory. Res. Synth. Methods.

[23] Rücker G., Schwarzer G. (2014). Reduce dimension or reduce weights? Comparing two approaches to multi-arm studies in network meta-analysis. Stat. Med..

[24] Dias S., Welton N., Caldwell D., Ades A. (2010). Checking consistency in mixed treatment comparison meta-analysis. Stat. Med..

[25] König J., Krahn U., Binder H. (2013). Visualizing the flow of evidence in network meta-analysis and characterizing mixed treatment comparisons. Stat. Med..

[26] Rücker G., Schwarzer G. (2015). Ranking treatments in frequentist network meta-analysis works without resampling methods. BMC Med. Res. Methodol..

[27] Molin L., Nyström B. (1970). A comparison between cephaloridine and penicillin in the treatment of gonorrhoea. Chemotherapy.

[28] Crider S.R., Kilpatrick M.E., Harrison W.O., Kerbs S., Berg S.W. (1984). A comparison of penicillin G plus a beta-lactamase inhibitor (sulbactam) with spectinomycin for treatment of urethritis caused by penicillinase-producing Neisseria gonorrhoeae. Sex. Transm. Dis..

[29] Veeravahu M., Sumathipala A., Clay J. (1983). Cefoxitin v procaine penicillin in the treatment of uncomplicated gonorrhoea. Br. J. Vener. Dis..

[30] Morrison G.D., Evans A.J., Haskins H.W., Lewis N.M., Seale G.H., Mayall E., Mullinger B.M. (1980). Cefuroxime compared with penicillin for the treatment of gonorrhea. Sex. Transm. Dis..

[31] Judson F., Ehret J., Root C. (1983). Comparative study of ceftriaxone and aqueous procaine penicillin G in the treatment of uncomplicated gonorrhea in women. Antimicrob. Agents Chemother..

[32] Judson F.N., Ehret J.M., Handsfield H.H. (1985). Comparative study of ceftriaxone and spectinomycin for treatment of pharyngeal and anorectal gonorrhea. JAMA.

[33] Handsfield H.H., Murphy V. (1983). Comparative study of ceftriaxone and spectinomycin for treatment of uncomplicated gonorrhoea in men. Lancet.

[34] Apaya J. (1984). Comparison of amoxycillin and procaine penicillin in the treatment of uncomplicated gonorrhoea. Br. J. Vener. Dis..

[35] Zajdowicz T., Sanches P., Berg S., Kerbs S., Newquist R., Harrison W. (1983). Comparison of ceftriaxone with cefoxitin in the treatment of penicillin-resistant gonococcal urethritis. Sex. Transm. Infect..

[36] Lossick J.G., Thompson S.E., Smeltzer M.P. (1982). Comparison of cefuroxime and penicillin in the treatment of uncomplicated gonorrhea. Antimicrob. Agents Chemother..

[37] Duanćić A., Fiumara N.J., Alpert S., Lee Y.-H., Tarr P.I., Rosner B., McCormack W.M. (1974). Comparison of spectinomycin hydrochloride and aqueous procaine penicillin G in the treatment of uncomplicated gonorrhea. Antimicrob. Agents Chemother..

[38] Baddour L.M., Busby L., Shapiro E., Cox K.B., Glassco S., Johnson J.K. (1992). Evaluation of treatment with single-dose ampicillin/sulbactam with probenecid or ceftriaxone in patients with uncomplicated gonorrhea. Sex. Transm. Dis..

[39] McCormack W.M., Mogabgab W.J., Jones R.B., Wendel J.G., Handsfield H. (1993). Multicenter, comparative study of cefotaxime and ceftriaxone for treatment of uncomplicated gonorrhea. Sex. Transm. Dis..

[40] Panikabutra K., Ariyarit C., Chitwarakorn A., Saensanoh C., Wongba C. (1985). Randomised comparative study of ceftriaxone and spectinomycin in gonorrhoea. Sex. Transm. Infect..

[41] Dixon C., Bittiner J., Shahidullah M., Slack R., Sulaiman M. (1986). Randomised observer blind comparative trial of ceftriaxone and penicillin in treating uncomplicated gonorrhoea in men and women. Sex. Transm. Infect..

[42] Mitchell R.W., Robson H.G. (1974). Single-dose treatment of gonococcal urethritis in males: Evaluation of procaine penicillin, ampicillin and spectinomycin. Can. Med. Assoc. J..

[43] Habte-Gabr E., Geyid A., Serdo D., Biddle J., Perine P.L. (1987). Single-dose treatment of uncomplicated acute gonococcal urethritis in Ethiopian men: Comparison of rosoxacin, spectinomycin, penicillin, and ampicillin. Sex. Transm. Dis..

[44] Pedersen A.H., Wiesner P.J., Holmes K.K., Johnson C.J., Turck M. (1972). Spectinomycin and penicillin G in the treatment of gonorrhea: A comparative evaluation. JAMA.

[45] Chitwarakorn A., Ariyarit C., Panikabutra K., Buateing A., Biddle J., Thompson S., Brown S. (1985). Treating gonococcal infections resistant to penicillin in Bangkok: Comparison of cefuroxime and spectinomycin. Sex. Transm. Infect..

[46] Simpson M., Khan M., Siddiqui Y., Gruninger R., Wigren D. (1981). Treatment of gonorrhea: Comparison of cefotaxime and penicillin. Antimicrob. Agents Chemother..

[47] Stolz E., Ong L., van Joost T., Michel M.F. (1984). Treatment of non-complicated urogenital, rectal and oropharyngeal gonorrhoea with intramuscular cefotaxime 1.0 g or cefuroxime 1.5 g. J. Antimicrob. Chemother..

[48] Hira S.K., Attili V.R., Kamanga J., Mkandawire O., Patel J.S., Patel M.I. (1985). Efficacy of gentamicin and kanamycin in the treatment of uncomplicated gonococcal urethritis in Zambia. Sex. Transm. Dis..

[49] Rajan V., Sng E., Thirumoorthy T., Goh C. (1982). Ceftriaxone in the treatment of ordinary and penicillinase-producing strains of Neisseria gonorrhoeae. Sex. Transm. Infect..

[50] Mogabgab W.J., Lutz F.B. (1994). Randomized study of cefotaxime versus ceftriaxone for uncomplicated gonorrhea. South. Med. J..

[51] Judson F.N., Allaman J., Dans P.E. (1974). Treatment of gonorrhea: Comparison of penicillin G procaine, doxycycline, spectinomycin, and ampicillin. JAMA.

[52] Boslego J.W., Hicks C.B., Greenup R., Thomas R.J., Wiener H.A., Ciak J., Tramont E.C. (1988). A prospective randomized trial of ofloxacin vs. doxycycline in the treatment of uncomplicated male urethritis. Sex. Transm. Dis..

[53] Lassus A., Karppinen L., Ingervo L., Jeskanen L., Reitamo S., Happonen H.-P., Karkulahti R. (1989). Ciprofloxacin versus amoxycillin and probenecid in the treatment of uncomplicated gonorrhoea. Scand. J. Infect. Dis Suppl..

[54] Scott G., McMillan A., Young H. (1987). Ciprofloxacin versus ampicillin and probenecid in the treatment of uncomplicated gonorrhoea in men. J. Antimicrob. Chemother..

[55] Dubois J., St-Pierre C., Olivier C., Clecner B., Austin T., Phillips R. (1990). Comparative Double-Blind Multicentre Study of Single-Dose Pefloxacin and Amoxicillin Plus Probenecid for Treatment of Acute Uncomplicated Gonorrhoea. Drug Investig..

[56] Karney W.W., Turck M., Holmes K.K. (1974). Comparative therapeutic and pharmacological evaluation of amoxicillin and ampicillin plus probenecid for the treatment of gonorrhea. Antimicrob. Agents Chemother..

[57] Gruber F., Brajac I., Jonjic A., Grubisic-Greblo H., Lenkovic M., Stasic A. (1997). Comparative trial of azithromycin and ciprofloxacin in the treatment of gonorrhea. J. Chemother..

[58] Roddy R., Handsfield H., Hook E. (1986). Comparative trial of single-dose ciprofloxacin and ampicillin plus probenecid for treatment of gonococcal urethritis in men. Antimicrob. Agents Chemother..

[59] Mitchell R.W., Robson H.G. (1974). Comparison of amoxicillin and ampicillin in single-dose oral treatment of males with gonococcal urethritis. Can. Med. Assoc. J..

[60] Felman Y.M., William D.C., Corsaro M.C. (1979). Comparison of ampicillin plus probenecid with amoxicillin plus probenecid for treatment of uncomplicated gonorrhea. Sex. Transm. Dis..

[61] Enfors W., Eriksson G. (1975). Comparison of oral ampicillin and doxycycline in the treatment of uncomplicated gonorrhoea. Sex. Transm. Infect..

[62] Brathwaite A. (1979). Double-blind trial of amoxycillin and ampicillin plus probenecid in the treatment of gonorrhoea in men. Sex. Transm. Infect..

[63] Jordan W.C. (1981). Doxycycline vs. Tetracycline in the Treatment of Men with Gonorrhea: The Compliance Factor.

[64] Black J., Long J., Zwickl B., Ray B., Verdon M., Wetherby S., Hook E., Handsfield H. (1989). Multicenter randomized study of single-dose ofloxacin versus amoxicillin-probenecid for treatment of uncomplicated gonococcal infection. Antimicrob. Agents Chemother..

[65] Cheong L., Chan R., Nadarajah M. (1992). Pefloxacin and ciprofloxacin in the treatment of uncomplicated gonococcal urethritis in males [corrected]. Sex. Transm. Infect..

[66] Megran D., Lefebvre K., Willetts V., Bowie W. (1990). Single-dose oral cefixime versus amoxicillin plus probenecid for the treatment of uncomplicated gonorrhea in men. Antimicrob. Agents Chemother..

[67] Karney W.W., Turck M., Holmes K.K. (1974). Single-dose oral therapy for uncomplicated gonorrhea: Comparison of amoxicillin and ampicillin given with and without probenecid. J. Infect. Dis..

[68] Jordan W.C. (1980). The efficacy of doxycycline vs tetracycline in treatment of gonorrhea in men. J. Natl. Med. Assoc..

[69] Lule G., Behets F., Hoffman I., Dallabetta G., Hamilton H., Moeng S., Liomba G., Cohen M. (1994). STD/HIV control in Malawi and the search for affordable and effective urethritis therapy: A first field evaluation. Sex. Transm. Infect..

[70] Baytch H., Rankin D. (1972). Comparison of penicillin, tetracycline, and doxycycline in the treatment of uncomplicated gonorrhoea in men. Br. J. Vener. Dis..

[71] Kirkcaldy R.D., Weinstock H.S., Moore P.C., Philip S.S., Wiesenfeld H.C., Papp J.R., Kerndt P.R., Johnson S., Ghanem K.G., Hook III E.W. (2014). The efficacy and safety of gentamicin plus azithromycin and gemifloxacin plus azithromycin as treatment of uncomplicated gonorrhea. Clin. Infect. Dis..

[72] Ross J.D., Brittain C., Cole M., Dewsnap C., Harding J., Hepburn T., Jackson L., Keogh M., Lawrence T., Montgomery A.A. (2019). Gentamicin compared with ceftriaxone for the treatment of gonorrhoea (G-ToG): A randomised non-inferiority trial. Lancet.

[73] Mann L.M., Kirkcaldy R.D., Papp J.R., Torrone E.A. (2018). Susceptibility of Neisseria gonorrhoeae to Gentamicin—Gonococcal Isolate Surveillance Project, 2015–2016. Sex. Transm. Dis..

